# Les reconstructions acétabulaires dans les prothèses totales de hanche

**DOI:** 10.11604/pamj.2015.22.225.6259

**Published:** 2015-11-10

**Authors:** Hassane Zejjari, Jamal Louaste, Taoufik Cherrad, Hicham Bousbae, Housseine Kasmaoui, Larbi Amhajji, Khalid Rachid

**Affiliations:** 1 Service de Chirurgie Traumatologique et Orthopédique de l'Hôpital Militaire Moulay Ismail de Meknès, Maroc

**Keywords:** Reconstruction acétabulaire, prothèse totale de la hanche, greffe osseuse, anneaux de soutiens, Acetabular reconstruction, total hip replacement, bone graft, support rings

## Abstract

La chirurgie de reconstruction acétabulaire est une technique qui consiste à combler les pertes de substance osseuse siégeant au niveau du cotyle. Il s'agit d'une étude rétrospective de 15 patients (16 cotyles), sur une période de 6 ans (2006- 2012). Dans douze cas il s'agissait de prothèse totales de la hanche de première intention et dans quatre cas il s'agissait de reprise cotyloïdienne de PTH. L’évaluation clinique préopératoire et postopératoire de tous nos patients a été effectuée par le score de Postel et Merle d'Aubigné. L’évaluation des pertes de substances osseuse du cotyle a été classée selon la classification de Paprosky. L’âge moyen de nos patients au moment de l'intervention a été de 57 ans avec des extrêmes allant de 20 ans à 76 ans. La greffe osseuse a été utilisée chez 12 de nos patients. La reconstruction prothétique a été utilisée chez 8 patients (anneau de Kerboul dans six cas et anneau de Burch-Schneider dans deux cas). Recul post opératoire moyen a été de 56 mois. L’évaluation radiologique a été basée sur les clichés radiologiques du bassin de face strict ainsi que des radiographies de la hanche opérée de face prenant la totalité de la prothèse. Nos résultats cliniques et radiologiques ont été jugé bon à très bon dans la majorité des cas. Pour nous, nous optant pour le recentrage-reconstruction car c'est lui qui s'approche le plus de l'anatomie et de la biomécanique normale de la hanche.

## Introduction

La chirurgie de reconstruction acétabulaire est une technique qui consiste à combler les pertes de substance osseuse siégeant au niveau du cotyle. Parfois considérables, Ces pertes de substances peuvent être cavitaires, segmentaires ou combinées. Le chirurgien doit alors faire un choix à la fois biologique et mécanique: soit placer la cupule proche du centre de rotation et combler la perte de substance osseuse, soit fixer la cupule en place sur l'os acétabulaire mais au prix d'une fréquente ascension du centre de rotation de la hanche. A travers cette étude rétrospective de 15 patients (16 cotyles), nous voulons exposer les résultats et les difficultés de cette reconstruction, tout en comparant nos résultats avec ceux de la littérature.

## Méthodes

**Figure 1:**
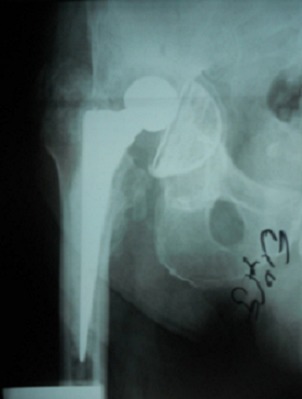
Descellement aseptique du cotyle avec luxation et protrusion de la cupule cotyloïdienne chez l'un de nos patients

**Figure 2:**
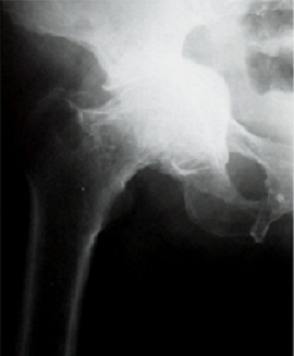
Coxarthrose protrusive primitive chez l'un de nos patients

**Figure 3:**
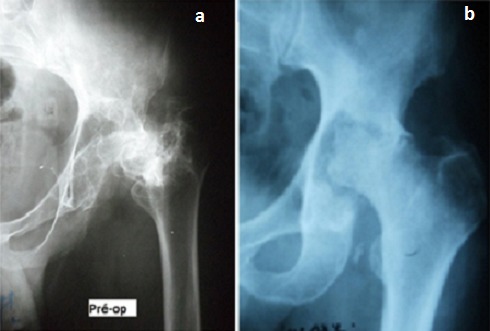
(a) Ostéonécrose tête fémorale chez un jeune patient suivi pour lymphome après sa cure de radiothérapie; (b) ostéonécrose du cotyle et de la tête fémorale secondaire à une radiothérapie pour cancer de la prostate

**Figure 4:**
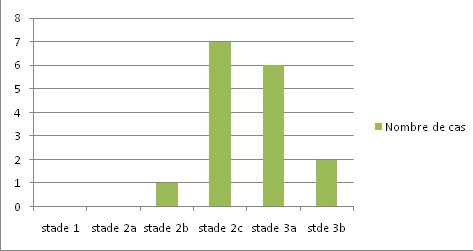
Répartition des pertes de substances osseuse du cotyle chez nos patients selon la classification de Paprosky

Notre étude porte sur les reconstructions du cotyle, au cours des Prothèses Totales de la Hanche (PTH). Dans douze cas il s'agissait de prothèse totales de la hanche de première intention et dans quatre cas il s'agissait de reprise cotyloïdienne de PTH. L’évaluation clinique préopératoire et postopératoire de tous nos patients a été effectuée par le score de Postel et Merle d'Aubigné. L’évaluation des pertes de substances osseuse du cotyle a été classée selon la classification de Paprosky. L’âge moyen de nos patients au moment de l'intervention était de 57 ans avec des extrêmes allant de 20 ans à 76 ans. Neuf de nos patients étaient des hommes et six étaient des femmes. Les patients ont été opérés du coté droit dans 9 cas, et, du coté gauche dans 5 cas. Une atteinte bilatérale a été notée dans un cas. Les indications ont été variables, un descellement acétabulaire dans quatre cas (Figure 1), une coxarthrose protrusive dans deux cas (Figure 2), une coxarthrose post-traumatique dans trois cas, une dysplasie acétabulaire, une coxite rhumatismale et une ostéonécrose dans deux cas chacune (Figure 3). La perte de substance osseuse, classée selon la classification de Paprosky, a montré une prédominance des stades 2c et 3 (Figure 4). Tous nos patients ont été opérés par voie postéro-externe de Moore. La greffe osseuse a été utilisée chez 12 de nos patients. Nous avons eu recours à des cupules cimentées dans 13 cas, une cupule cimentée double mobilité dans un cas et des cupules non cimentée dans deux cas. Les anneaux de soutien ont été utilisés dans huit cas. Il s'agissait d'un anneau de Kerboul dans six cas et d'un anneau de Bursch-Schneider dans deux cas.

## Résultats

**Figure 5:**
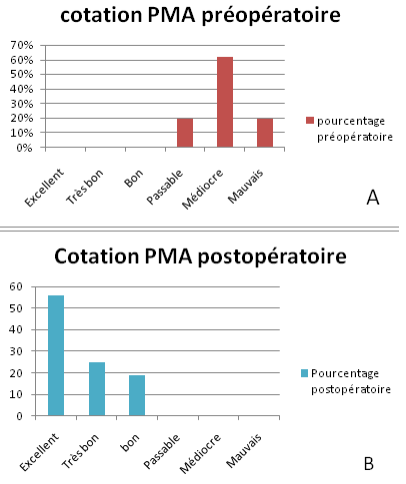
L’évolution de la cotation PMA de nos patients: (A) en préopératoire; (B) en postopératoire

**Figure 6:**
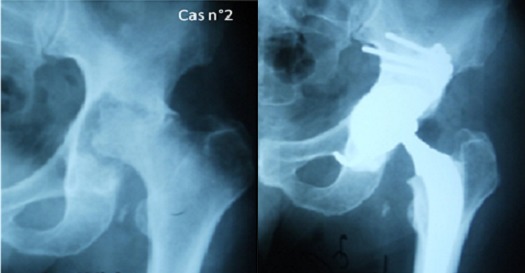
Radiographies pré et postopératoire chez l'un de nos patients montrant un bon centrage de la hanche reconstruite

**Figure 7:**
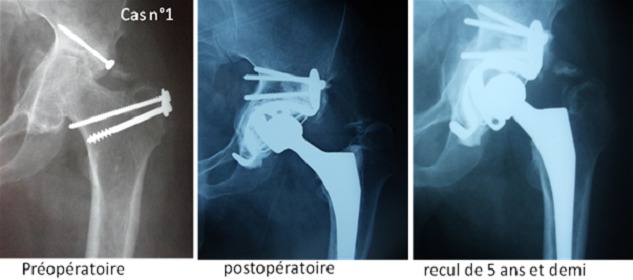
Radiographies de la hanche chez l'un de nos patients montrant la bonne intégration de la greffe osseuse

Recul post opératoire moyen a été de 56 mois, avec des extrêmes allant de 12 à 72 mois. Les résultats fonctionnels des hanches opérées selon la cotation de Postel et Merle d'Aubigné ont montrée une nette amélioration par rapport aux chiffres préopératoires (Figure 5). Aucun cas d'infection précoce ou de complication thromboembolique n'a été noté. Deux épisodes de luxations ont été rapportés chez la patiente opérée pour séquelles de fractures des 2 cotyles qui n'ont pas nécessitées de reprise. L’évaluation radiologique a été basée sur les clichés radiologiques du bassin de face strict ainsi que des radiographies de la hanche opérée de face prenant la totalité de la prothèse. L’étude soigneuse des radiographies successives et leur confrontation avec le cliché post opératoire précoce, constituent le temps principal de la surveillance de toutes les arthroplasties totales de la hanche qu'on a implantées afin de dépister des complications débutantes et de proposer une réintervention. Dans un souci de simplicité l’étude radiologique a été réalisée grâce aux trois radiographies de bassin de face: pré, postopératoire et au dernier recul. Le positionnement de l'implant a été évalué par la classification de la Société Orthopédique de l'Ouest (SOO 2004). L'inclinaison de l'implant sur l'horizontale a été mesurée par rapport à la ligne bi-ischiatique. Le positionnement vertical et horizontal de l'implant a été jugé correct dans toutes les hanches de la série (c'est-à-dire en zone II B de la classification de la SOO) (Figure 6). L'inclinaison moyenne de la cupule était en moyenne de 43,75°, et l'inclinaison moyenne de l'anneau était de 50,87° en postopératoire; Ce positionnement a été stable dans le temps pour toutes les hanches étudiées; ainsi, les variations de position horizontale ou verticale du centre de la cupule étaient <3 mm dans 100% des cas. La correction moyenne du raccourcissement préopératoire était de 11,8 mm (minimum -6 mm, maximum 33 mm). Aucune ossification péri-prothétique n'a été observée. L'intégration de la greffe osseuse a été jugée bonne chez tous nos patients au dernier recul (Figure 7).

## Discussion

La chirurgie de reconstruction acétabulaire est une technique qui consiste à combler les pertes de substance osseuse siégeant au niveau du cotyle. Les objectifs en terme de reconstruction acétabulaire sont la restauration du stock osseux par l'utilisation de greffe osseuse autologue ou non, la restauration du centre de rotation anatomique de la hanche par l'utilisation des anneaux de soutien et enfin assurer une stabilité primaire satisfaisante de la cupule, surtout des cupules non cimentées. La reconstruction acétabulaire nécessite de définir, d'analyser et de classer de façon reproductible la perte de substance osseuse afin de se donner les moyens para-cliniques d'une planification préopératoire. Qualitativement, le toit, la paroi antérieure, la paroi postérieure, l'arrière-fond et l’échancrure peuvent être atteints. La perte de substance peut être segmentaire, cavitaire ou mixte [1, 2]. Une perte de substance segmentaire se définit comme une interruption complète, ou incontinente de l'anneau acétabulaire ou de la paroi médiale de l'acétabulum. Une perte de substance cavitaire est volumétrique et continente, sans interruption de continuité de l'anneau. Quantitativement, la perte de substance est évaluée en fonction de son extension selon un cadran horaire et en fonction de ses dimensions réelles mesurées en centimètre. Cette mesure peut être complétée par celle du pourcentage d'appui hémisphérique du futur implant sur l'os receveur [3, 4]. L’évaluation per-opératoire de la perte de substance acétabulaire doit faire appel à l'association de toutes ces méthodologies descriptives. L'objectif est d'aboutir à une classification fiable et reproductible nécessaire à la prise en charge chirurgicale. La classification de D'Antonio [1, 2] a introduit la notion de perte de substance segmentaire et cavitaire, centrale et/ou périphérique. La classification de Paprosky [5] se veut plus pragmatique, basée sur la capacité de l'anneau acétabulaire à fournir un support mécanique suffisant pour la nouvelle cupule. Elle sous-entend donc une notion de stratégie de reconstruction. La classification de Gross [3, 6] reprend la description de perte de substance segmentaire et cavitaire avec un appui supérieur ou inférieur à 50%. Enfin la classification française de Vives [7] est plus simple avec quatre stades de gravité croissante. Perrier et Gouin [8] ont étudié la validité préopératoire de ces quatre classifications en se basant sur un bilan radiographique orthogonal standard. L’étude a porté sur 33 dossiers de reconstructions acétabulaires réalisées chez 31 patients opérés au CHU de Nantes entre le 1erjanvier 1996 et le 31 décembre 2000. Deux analyses de la perte de substance ont été effectuées par 6 observateurs d'expérience différente à 15 jours d'intervalle. Ils ont évalué la concordance intra et inter-observateur et l'existence d'une différence statistique entre les évaluations pré et peropératoires. Ils ont ainsi obtenu des concordances inter et intra-observateur négligeables à modérées, ainsi qu'une absence de relation statistique entre les données préopératoires et peropératoires. Ce résultat, confirmé par les données récentes de la littérature internationale [9, 10], montre que l’établissement préopératoire de ces classifications ne permet pas d’évaluer de manière fiable les pertes de substances retrouvées en peropératoire. Lors de l'enquête de pratique SOO, 58% seulement des 151 chirurgiens utilisaient une classification préopératoire et pour les deux tiers d'entre eux la plus simple, c'est-à-dire, celle de la SOFCOT. La planification 1D doit faire partie du bilan préopératoire. Les classifications se montrent difficilement utilisables car elles mélangent des critères pré et peropératoires. Elles pourraient être remplacées par l’évaluation du centrage de la cupule descellée sans doute plus reproductible. Elle ne dispense toutefois pas de la description de la perte de substance dans nos comptes-rendus opératoires, en précisant leurs topographies, leurs étendues, leurs caractères segmentaires et cavitaires ainsi que le pourcentage d'appui de l'implant.

Dans notre série nous avons opté pour la classification de Paprosky, vu que c'est la plus utilisée dans la littérature. La TDM doit être considérée comme un complément non systématique des radiographies standards car elle représente un coût supplémentaire et un surcroît d'irradiation qui ne se justifie pas lorsque les incidences standards de face et oblique ont été suffisamment informatives. A l'inverse dans certains cas d'analyse difficile pour le diagnostic positif de descellement cotyloïdien ou pour la caractérisation et la localisation de la perte de substance osseuse, il vaut certainement mieux réaliser un scanner avec des reconstructions multi-planaires plutôt que de multiplier les incidences radiologiques, elles aussi irradiantes [11]. L'objectif primaire de la reconstruction acétabulaire est de redonner au patient une hanche indolore, stable avec une fonction de hanche satisfaisante. Il peut se décliner en quatre objectifs: l'implantation d'un implant bien fixé et stable dans le temps, la restauration du centre de rotation et de la longueur du membre inférieur, l'obtention d'une bonne stabilité de la hanche. Différents moyens s'offrent pour réaliser la reconstruction osseuse de l'acétabulum. Ces moyens diffèrent selon leur nature, autogreffe, allogreffe morcelées, structurales ou massives, biomatériaux et substituts osseux. Leurs indications respectives dépendent du type de perte de substance osseuse observée, segmentaire ou cavitaire. Les résultats sont liés à leur potentiel d'ostéointégration ou au contraire de résorption, eux-mêmes directement corrélés au type d'implant qui leur est associé, cimenté ou non, avec soutien ou non. L'autogreffe [12–15] constitue le Gold Standard des moyens de reconstruction osseuse. Elle peut être utilisée sous forme de greffons spongieux prélevés sur la crête iliaque antérieure ou postérieure. Elle est toutefois limitée par les quantités disponibles, surtout dans les pertes de substance volumineuses. Elle peut toutefois dans ces cas là être associée à une allogreffe dans le but d'en améliorer l'ostéointégration. L'autogreffe peut également être utilisée sous forme de greffon cortico-spongieux pour reconstruire un défect segmentaire. Tabutin a décrit la greffe en palissade qui permet de reconstruire un défect supérieur par un greffon iliaque tricortical encastré et de recentrer la hanche en y associant une cupule impactée [16]. Leurs inconvénients majeurs sont la faible quantité disponible et la morbidité liée au prélèvement. Les allogreffes représentent le matériel le plus utilisé pour les reconstructions acétabulaires. Elles comportent trois formes disponibles: les allogreffes structurales, les allogreffes morcelées et les allogreffes massives. Elles sont d'origine humaine et peuvent être cryoconservées, irradiées ou lyophilisées. Elles peuvent être encastrées ou fixées par des vis ou une plaque d'ostéosynthèse [17, 18]. Paproski et Magnus préconisent l'utilisation d'allogreffes de fémur distal plutôt que de têtes fémorales [19, 20]. La plupart des études observent une consolidation plus constante avec les greffons morcelés qu'avec les greffons structuraux, plus sujets à des phénomènes de résorption [21]. Sloof a décrit initialement la technique de compactage des allogreffes morcelées avec cimentage direct de la cupule dans le lit de greffons impactés [22]. La survie à 15ans est de 84% mais le critère d’échec est la révision de la cupule, excluant donc les images radiographiques défavorables [23]. Cette technique a également été utilisée en interposant un anneau de renforcement entre les greffons et la cupule cimentée. Selon la série initiale de Zehntner et Ganz [24], les greffons impactés avec l'anneau de Müller ont tendance à se tasser. Avec l'anneau de Müller-Ganz, les résultats rapportés par Gerber et al sont meilleurs avec un taux de survie à 10 ans de 81%.

Cinq patients sur 46 suivis ont présenté un descellement itératif [25]. Au total, à la synthèse de ces résultats, les greffons tassés morcelés doivent probablement être utilisés avec la technique initialement décrite par Slooff c'est-à-dire avec cimentage direct dans les greffons impactés. En ce qui concerne les allogreffes structurales en zone portante, il semble qu'il soit préférable de les armer par un anneau de renforcement acétabulaire comportant un appui inférieur, dont l'exemple le plus connu est la croix de Kerboull avec son crochet inférieur. Enfin, lorsque la taille de l'allogreffe est importante (plus de 50% de la zone portante), il est préférable d’éviter la fixation sans ciment. Ces tendances sont confirmées par les résultats récemment publiés du registre norvégien des révisions acétabulaires [26]. Quoi qu'il en soit, dans l'ensemble des séries publiées, l'ossification des allogreffes apparaît inconstante, comme le montre l'observation radiographique de tassements au-delà de 10 ans. Le ciment acrylique ou polyméthylmétacrylate (PMMA) est le biomatériau le plus ancien utilisé dans la reconstruction du cotyle mais surtout dans les révisions acétabulaires de PTH. Il constitue un moyen simple de comblement mais avec une qualité de fixation relativement aléatoire sur un os de mauvaise qualité souvent scléreuse. Il ne permet toutefois pas une reconstruction du capital osseux, expose à un descellement itératif et doit pratiquement être abandonné en tant que matériau de comblement osseux surtout dans les révisions acétabulaires [7]. Les implants disponibles pour effectuer une reconstruction acétabulaire sont multiples et de nombreux systèmes ont été développés pour permettre une fixation stable même dans les défects osseux importants. Le choix de l'implant sera lié à l'importance du défect, à la surface de l'os hôte pouvant supporter les contraintes de l'implant et au type de reconstruction osseuse utilisé. L'existence d'une pseudarthrose peut nécessiter l'association d'une ostéosynthèse par plaque vissée pour assurer au montage une stabilité satisfaisante. Ces types d'implants sont répartis en trois catégories: les cupules primaires, les anneaux de soutien, les cupules de grand diamètre et les cupules modifiées. Les anneaux de soutien sont des implants métalliques que l'on fixe par des vis spongieuses dans l'os coxal et dans lesquels on scelle une cupule qui peut être une cupule de polyéthylène (PE), une cupule de PE a intérieur métal pour un couple de friction métal-métal, ou encore une cupule à double mobilité pour réduire les risques de luxation. Ces anneaux de soutien permettent d'améliorer la fixation et le centrage de la cupule, de répartir l'appui sur le pourtour de l'acétabulum, de compenser éventuellement une perte de substance osseuse segmentaire peu étendue, d'assurer la fixation des allogreffes et éventuellement de réaliser l'ostéosynthèse d'une pseudarthrose ou discontinuité pelvienne. Le scellement de la cupule dans l'anneau de soutien peut se faire avec une orientation différente de la position de l'anneau, ce qui permet d'avoir une position optimale et stable de la reconstruction par l'anneau et également une position optimale de la cupule pour assurer une bonne stabilité de la hanche [27]. L'anneau de soutien de Muller a un appui périphérique; il permet une fixation par vissage endo-acétabulaire et périphérique surtout dans le toit et la colonne postérieure mais ne peut seul assurer l'ostéosynthèse d'une fracture de l'acétabulum. Il est recommandé d'avoir un contact supérieur, postérieur et inféro-médial [28]. Garbuz souligne le caractère fondamental d'une fixation par les vis dans l'os receveur [29].

L'anneau de soutien permet de protéger l'allogreffe et ainsi d’éviter la migration supérieure de l'implant et l'incidence des descellements aseptiques [30, 31]. Les anneaux à crocher obturateur sont principalement représentés par l'anneau de Ganz [25] et par l'anneau de Kerboull (ou croix de Kerboull) [32]. Ils permettent un meilleur centrage de la cupule. La croix de Kerboull dispose en outre d'une palette supérieure qui est vissée dans la partie basse de l'aile iliaque, ce qui nécessite toutefois un abord un peu plus extensif. Ces systèmes permettent en théorie de traiter une fracture associée de l'acétabulum mais avec une fixation un peu précaire lorsqu'ils sont utilisés seuls [17]. Par son crochet la croix de Kerboull permet un recentrage automatique de la hanche [32]. Dans une étude Melchior et al. retrouvent un centre de rotation en zone optimale dans 88% des cas. La croix est suffisamment rigide pour assurer la fixation d'une discontinuité du bassin mais sollicite néanmoins probablement les greffons. Dans cette étude un taux de migration radiologique de 10% à 7 ans a été noté, avec une résorption partielle ou totale de la greffe osseuse. En comparaison, le taux de survie dans la série de Kerboull [32], était de 92% à 13 ans. Il n'est pas noté de dégradation tardive des résultats, dans cette étude à long terme. Les anneaux anti-protrusion sont essentiellement représentés par l'armature anti-protrusion de Bursch-Schneider (AAP de B-C). Elle comporte une palette supérieure prenant appui sur l'aile iliaque et une patte inférieure prenant appui dans l'ischion. Elle permet de stabiliser une fracture de l'acétabulum, une protection de la greffe osseuse, une restauration du centre de rotation de la hanche et un pontage des grandes pertes osseuses causant une discontinuité pelvienne. Wachtl et al. [33] rapportent 38 révisions acétabulaire en utilisant AAP de B-C avec un recul moyen de 12 ans. Ils ont conclu que les résultats obtenus par cet anneau de soutien et comparables aux autres implants. Koevringe et Ochner [3] ont utilisé AAP de B-C chez 31 patients leurs résultats après un recul moyen de 5 ans ont été satisfaisants. Les cupules de grand diamètre (jumbo cup) sont des cupules non cimentées impactées de diamètre supérieur à 65 mm, pouvant aller jusqu’à 80 mm. Elles ont l'avantage de permettre de traiter des pertes de substances importantes sans nécessiter de reconstruction osseuse complexe par greffe [34]. Elles présentent en revanche l'inconvénient de modifier souvent la position du centre de rotation de la hanche, de sacrifier parfois la colonne antérieure en raison de la différence entre le diamètre antéro-postérieur et le diamètre proximo-distal, et de pouvoir favoriser un conflit antérieur avec le psoas en cas de débord antérieur [33, 35]. Cette technique constitue toutefois une escalade prothétique qui, en cas de descellement itératif, confronte à une perte de substance plus grande encore. Pour cette raison, cette technique n'est envisageable que chez des sujets âgés ou fragiles, dont l'espérance de vie permet de penser qu'il n'y aura pas chez eux d'autre indication de révision de la PTH. Des cupules bilobées, de forme oblongue, ont été développées pour s'adapter aux défects supérieurs. En effet, les pertes de substance intéressent souvent le toit et confèrent ainsi à l'acétabulum une forme ovale, oblongue. Ces implants permettent ainsi de combler le défect et de repositionner le centre de rotation de la hanche, de façon assez simple et rapide, sans nécessiter de greffe [36, 37]. Certains de ces implants sont modulaires par adjonction de cales ou d’«augments» de formes et de dimensions variables pour s'adapter aux défects [38].

## Conclusion

La reconstruction acétabulaire impose une planification préopératoire soigneuse. Le compte-rendu opératoire doit décrire l’état anatomique des structures acétabulaires portantes. L'utilisation d'une classification préopératoire est difficile et peu reproductible. Cet inconvénient peut être pallié par une classification basée sur des mesures et non des descriptions. Dans la revue de littérature les auteurs n'ont toutefois pas permis de départager clairement recentrage-reconstruction et fixation en place, aussi bien en termes de stabilité musculaire que de pérennité de la fixation de la cupule. Pour nous, nous optant pour le recentrage-reconstruction car c'est lui qui s'approche le plus de l'anatomie et de la biomécanique normale de la hanche.
